# The ins and outs of errors in oncology imaging: the DAC framework for radiologists

**DOI:** 10.3389/fonc.2024.1402838

**Published:** 2024-10-04

**Authors:** Antoine Iannessi, Hubert Beaumont, Carlos Aguillera, Francois Nicol, Anne-Sophie Bertrand

**Affiliations:** ^1^ Diagnostic and Interventional Radiology Department, Cancer Center Antoine Lacassagne, Nice, France; ^2^ Median Technologies, Imaging Lab Research Unit, Valbonne, France; ^3^ Clinical Research Department, Therapixel Research Unit, Nice, France; ^4^ Neuromod Institute , Centre Mémoire, Institut Claude Pompidou, Nice, France; ^5^ Imaging Department, Imaging Center, Beaulieu-sur-mer, Beaulieu-sur-Mer, France

**Keywords:** diagnostic errors/statistics and numerical data, radiologists, cognition, scientific mistake, quality improvement, oncology, risk factors, tomography mammography

## Abstract

With the increasingly central role of imaging in medical diagnosis, understanding and monitoring radiological errors has become essential. In the field of oncology, the severity of the disease makes radiological error more visible, with both individual consequences and public health issues. The quantitative trend radiology allows to consider the diagnostic task as a problem of classification supported by the latest neurocognitive theories in explaining decision making errors, this purposeful model provides an actionable framework to support root cause analysis of diagnostic errors in radiology and envision corresponding risk-management strategies. The D for Data, A for Analysis and C for Communication are the three drivers of errors and we propose a practical toolbox for our colleagues to prevent individual and systemic sources of error.

## 1 Introduction

According to an annual complaints report for radiologists in France, “medical error” affected 1 in 20 radiologists. A similar US analysis of complaints analysis filed over one decade showed that oncology errors accounted for the largest proportion accounting from 40% up to 80% of complaints when focusing on complaints with high harm (1, 2).

In this context, it is interesting to explore the mechanisms of “radiological error” by looking more specifically at this most represented field, i.e., cancer diagnostic radiology Mammography and computed tomography were the most concerned modalities probably due to the breast cancer screenings over the world and widespread use of CT scans for generic whole-body analysis (3). Ultrasound, even though it is frequently indicated as a first line examination was not predominantly concerned maybe due to the impossible retrospective analysis unlike other acquired modalities that can be objectively reassessed by a second opinion.

Previous frameworks for root-causing radiological errors have been proposed and the most used classification was published in 2014 by Kim and Mansfield (4). However, its applicability in terms of risk management is not straightforward. Radiological errors being primarily human errors, we propose to update a classification in accordance with the latest neurocognitive knowledge to regroup by underlying concepts and mechanics.

In this paper, firstly, we explore the terminologies of error in experimental medicine together with the current critical place of quantitative imaging in oncology. Then, we detail cognitive mechanisms of decision-making in radiology as applied to oncology to propose an updated error classification supported by signal detection theory. Finally, we detail strategies for managing the risk of error at both the individual and systemic level.

## 2 The” experimental” medicine and the concept of biomarkers

### 2.1 The “error” terminology

**Figure 1:**
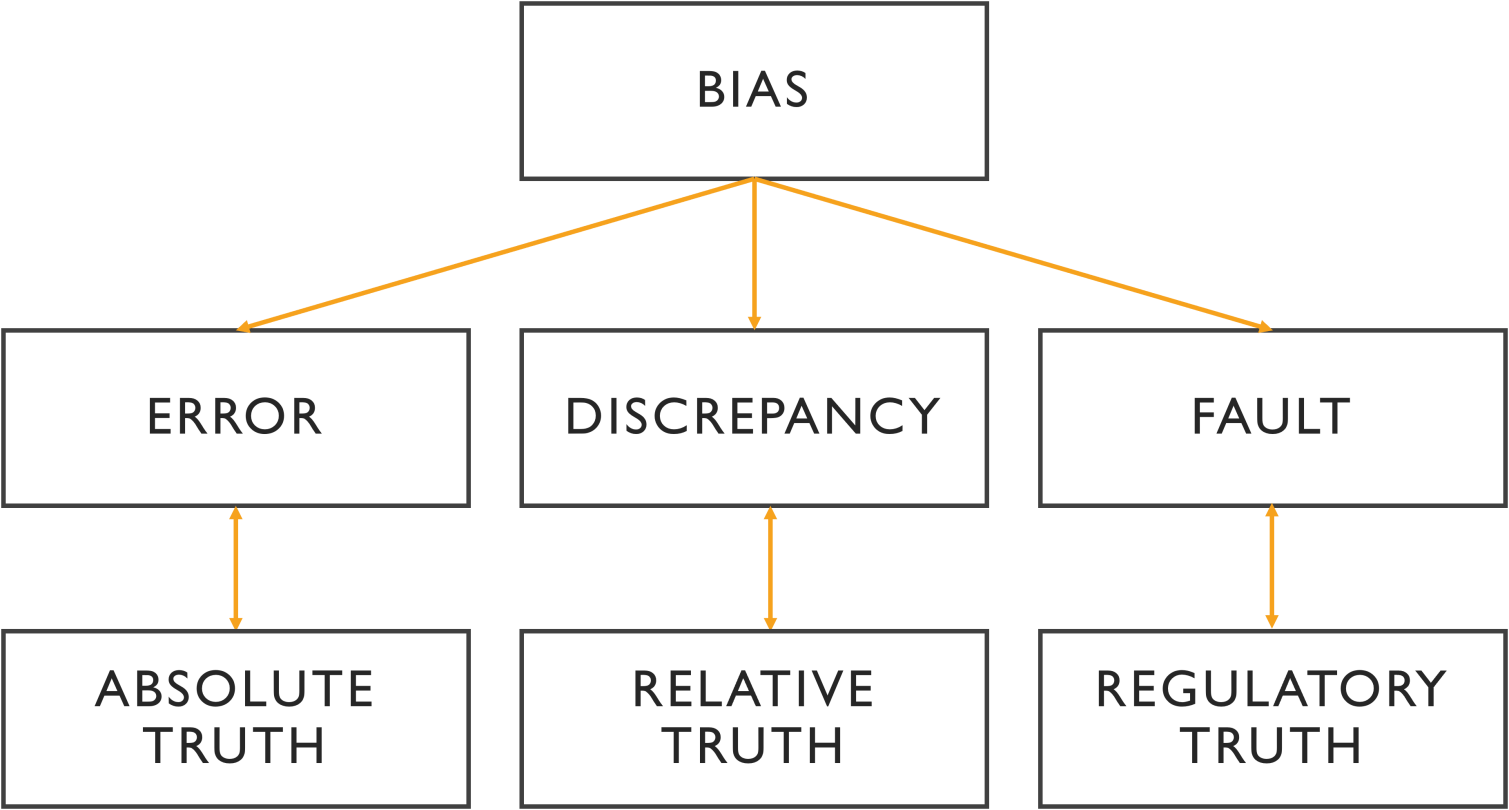
Relationship between the different terminologies of medical error. In the absence of intangible ground truth, the variability of analysis is reflected in discrepancy, where the benchmark can be a third expert opinion or a consensus. A fault is a type of error with a legitimate basis referring to non-compliance with the commonly accepted guidelines. More than an error, bias is a cause of error that is often systematic and unidentified.

The terminology related to “error” is deeply embedded in the history of experimental and quantitative medicine. This terminology is critical for understanding scientific literature and for comparing results across different studies (**Figure 1**).

However, depending on the field -whether in statistics or forensic medicine- the same terms can carry different meanings, often leading to confusion. A frequent source of this confusion is the distinction between “error” and “variability (5, 6). This issue dates back to the early days of quantitative experimentation, which were influenced by Mendel’s mechanistic approach to biology and his application of mathematical principles.

In the context of image analysis, variability refers to differences in interpretation between multiple observers. This is typically measured through indices that reflect the level of agreement or discordance among radiologists. These indices are essential for evaluating the reliability of diagnostic decisions (7).

Since error implies that what is correct is known, the notion of “truth” is inseparable from the notion to which error refers. In the era of quantitative medicine, we have inherited the term “ground truth” or “gold standard”, which is defined by empirical evidence, i.e., information given by direct sensory or experimental observation that is known to be true. This idea of truth aligns with the philosophical notion of “a posteriori knowledge,” knowledge that is based on experience, as opposed to “*a priori* knowledge,” which is derived from reasoning alone (8).

The relationship between error and experience is fundamental to evidence-based medicine, where decisions are grounded in both experimental results and practical experience (9).

In legal contexts, the term “fault” is used as a type of error, but with a critical distinction: an error is considered a fault only when the reference of the correctness is indisputably known in advance. In radiology, this typically means adherence to established best practices (10, 11).

In statistical and cognitive science, we encounter the term “bias”, which is a systematic cause of error that is mostly unconscious or invisible and *a priori*. A cognitive bias specifically refers to errors in thinking caused by a distortion in how information is processed.

While these terms -error, variability, fault, and bias- are distinct, they are also interrelated. **Figure 1** visually represents the relationships between these concepts and their importance across medical, statistical, and cognitive domains.

### 2.2 Quantitative imaging as a decision-making tool in cancerology

Medical imaging is a decision-making tool for the cancer patient that not only has to be performant but must also be used properly and at the right time to be efficient.

#### 2.2.1 Imaging biomarkers

The term “imaging biomarker” defines discriminating information contained in a medical image (12, 13). This information is measured by imaging modality and must satisfy characteristics that allow it to be used for decision-making, i.e., accuracy, precision, reliability, and relevance (14). The first three characteristics are also quantifiable. In addition, when studying “error” in radiology, it is essential to continually evaluate the source of information for its relevance and biological plausibility within the clinical context.

#### 2.2.2 Oncology and surrogate endpoints

**Figure 2:**
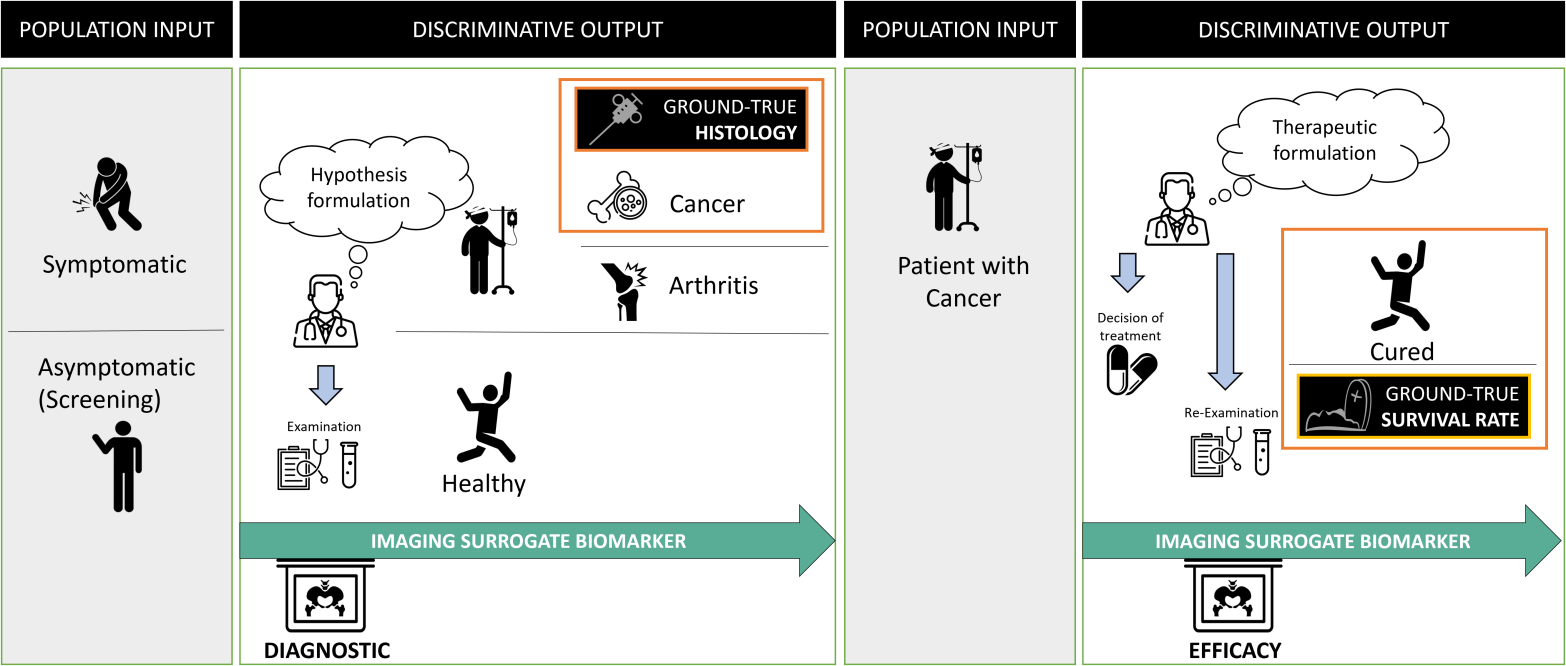
Problem statement in oncology and surrogate imaging biomarkers. Radiological diagnosis is over-simplified as a classification problem. In oncology, the statement of this problem evolves over time and requires discriminating between different populations at entry and exit. At the initial diagnostic stage, the aim is to discriminate between diseased (in which the cancer) and non-diseased patients. The imaging is compared with the gold standard, usually, invasive procedure to obtain the histology of the cancer. Following diagnosis, the discriminative test must recognize patients not responding to the treatment to offer them a better alternative. At this stage, the gold standard for judging the outcome is the death rate and one assumes that imaging biomarker can be a surrogate predictor of the overall survival rate.

The oncologic context provides a good example of “*a priori* discriminating” use of biomarkers (**Figure 2**). Their use differs depending on cancer stage. It is either a diagnostic question in a symptomatic or asymptomatic patient (i.e., during screening), or a question of the cancer treatment’s efficacy (i.e., during follow-up).

In both cases, the ground truth can only be known a posteriori since it corresponds to histology or surgery (for disease diagnosis), or death (for treatment efficacy).

Oncology imaging biomarkers are “surrogates” endpoints when they can be used instead of clinical outcomes. Indeed, invasive diagnosis is morbid, and death is fatal, therefore imaging biomarker-assisted decision-making attempts to predict disease or treatment ineffectiveness as early as possible with minimal error. The validity of such surrogates is regulatory and based on previously acquired evidence (15).

## 3 Cognitive decision model in cancer imaging

The decision step turns a virtual error into a real, and potentially harmful entity. Therefore, there is a notion of risk in making a decision and since the impact is substantial when considering oncology imaging, the stakes of this risk are higher.

To understand errors, it is essential to understand how both the tools (i.e., quantitative imaging biomarkers) and context of the decision-making (surrogates) described above are integrated by the individual operator to take his decision.

Modern cognitive science theories allow us to better understand the brain mechanism underlying decision making in these situations of uncertainty.

“Signal theory” and the Bayesian model represent the state-of-the-art of understanding of the decision mechanism, from the sensorial information to the decisional action (16, 17).

**Figure 3:**
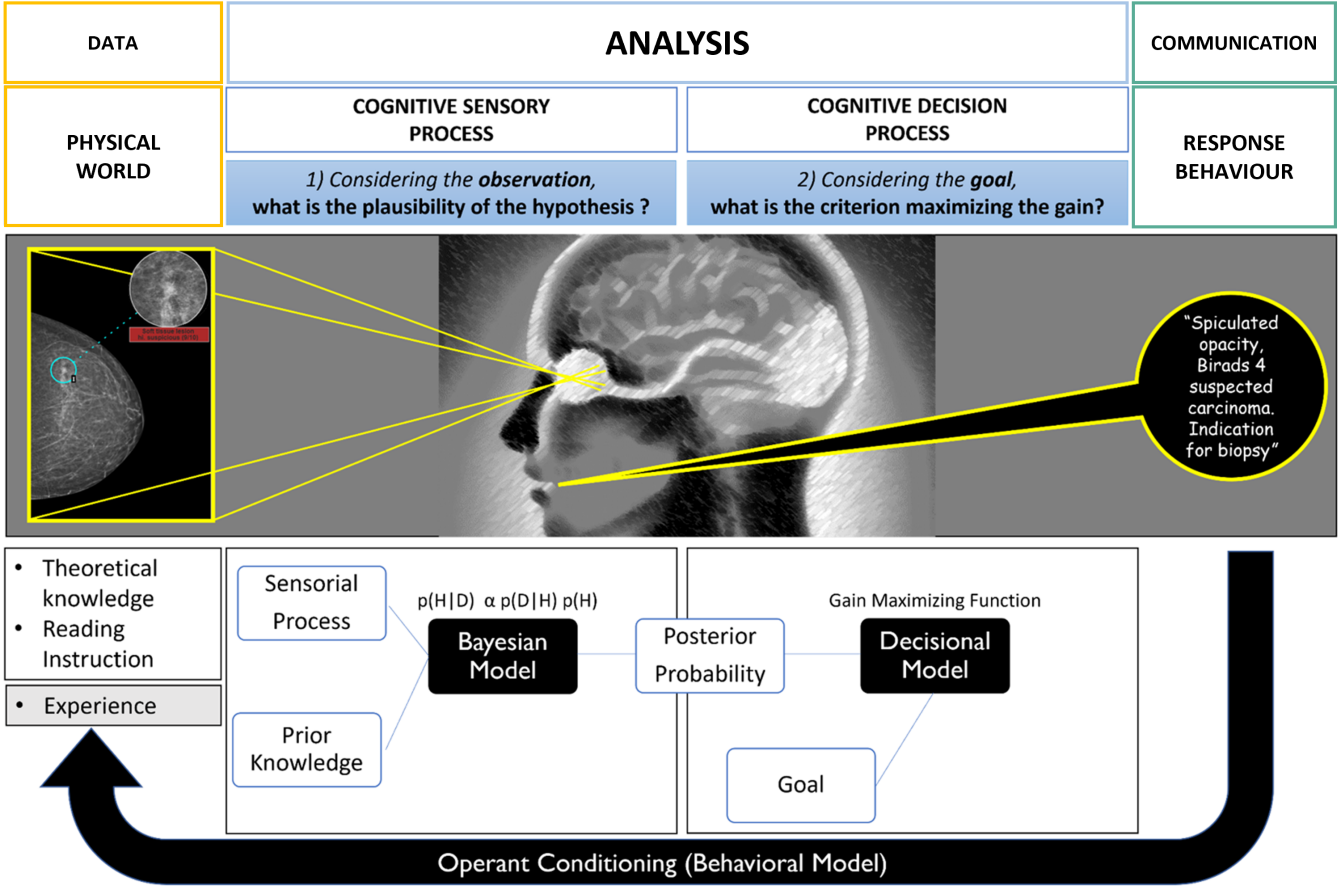
Cognitive modeling of decision-making during mammography screening (inspired by the theoretical model of decision in complex situations). The first sensory step is based on the Bayesian law of probabilities and produces plausible diagnostic hypotheses. The second step of the process is decisional and integrates the risk of error by choosing a decision criterion to separate the different hypotheses. Ultimately, there is an experimental feedback loop of success/error which influences the “a priori” knowledge for future diagnoses.

To best illustrate schematically the mental stages during the radiological diagnosis process, we consider the mammographic screening use case (**Figure 3**).

To address this task, the mental activity asks two sub questions which, for the sake of understanding, are represented sequentially but are in fact intertwined i.e.:

### 3.1 “Given what I see on the mammogram, how plausible is the hypothesis that my patient has cancer?”

In a probabilistic Bayesian model, this “plausibility” is denoted by p(H|D) and corresponds to the probability of having cancer (H) knowing the mammographic information (D). Conversely, the “a posteriori probability of having this mammographic data knowing the hypothesis”, is noted p(D|H) and corresponds to the “likelihood” of the data.

The law of probabilities shows that the plausibility of a given hypothesis is a function of the likelihood but also the “*a priori* probability” of the hypothesis noted p(H). This so-called “prior” contextualizes the decision making and greatly modifies the outcome. The law is written as:


p(H|D)∝p(D|H)×p(H)

It can be well understood by general radiologists that have experienced lung parenchymal findings on a coughing patient with a known evolutive cancer. They know that this may correspond to either a metastasis or an inflammation suggesting that the likelihood of the image alone is not discriminating enough. It is the *a priori* knowledge that allows us to conclude about plausibility (18).

In our practice, this “prior” corresponds to theoretical and experiential knowledge, to the context of the analysis and to the instructions for performing the task (i.e., reading the images) that is related to it. In oncology, for example, the latter read rules correspond to the BIRADS analysis for the mammography or RECIST criteria for an oncologic follow-up. Those rules are framing the context of the task (19).

Also, in processing this first sub question, there are two irreducible variability factors to consider, one concerning the data i.e., imaging biomarker and its signal/noise ratio (external noise), the second concerning the performance of the radiologist observer (internal sensory noise) which is equivalent to a “detector” intrinsic performance (20). The latest participates both in inter-observer variability and intra-observer variability as it varies for a same individual according to mentally perturbating stimuli (e.g., tiredness, stress, external stimuli).

### 3.2 Given the purpose of the mammography exam, what criterion maximizes its gain?

**Figure 4:**
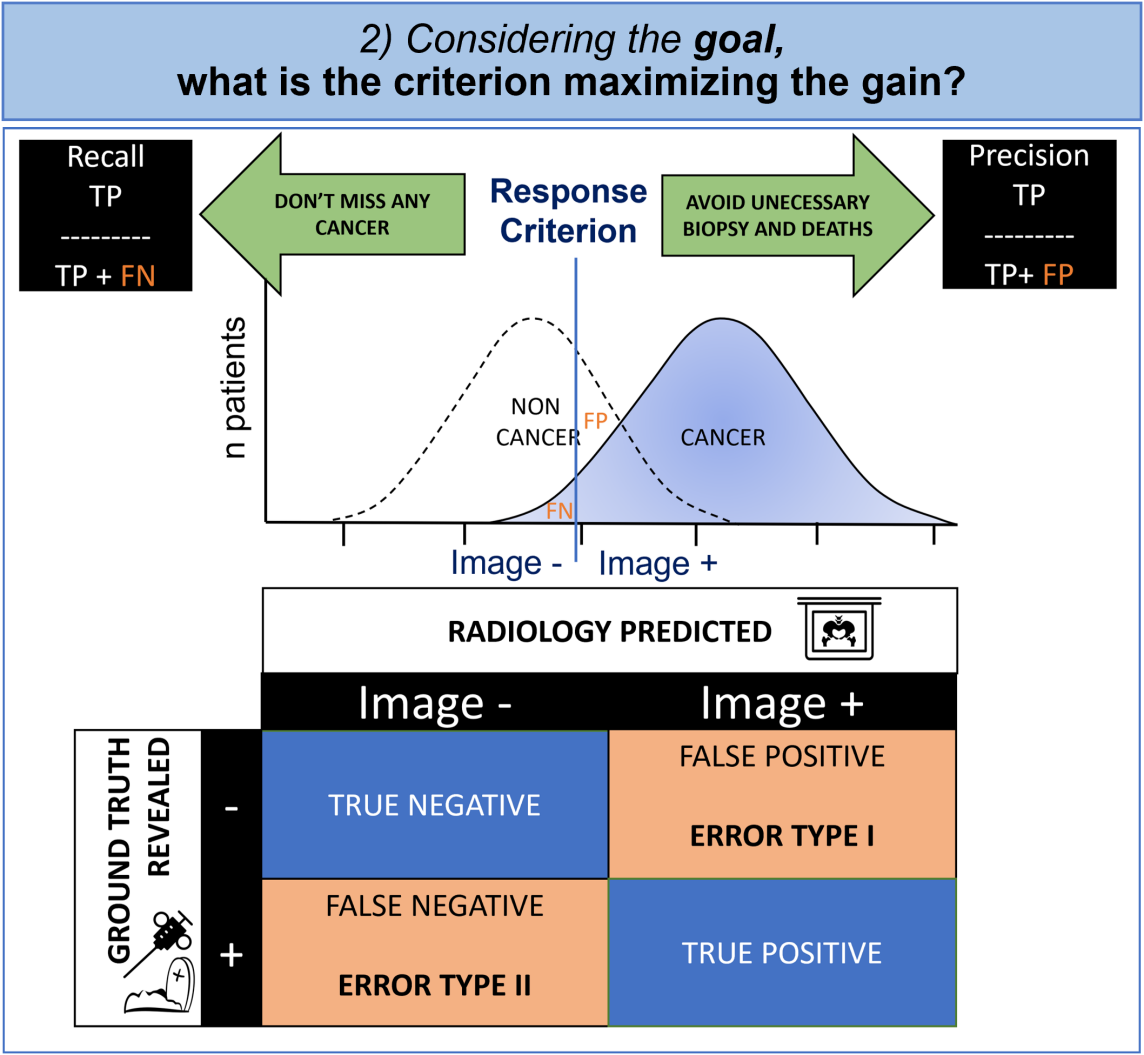
Cognitive modeling of decisional step: contingency table and criterion. Due to the overlapped distribution of probability in healthy patients and those with cancer, the radiologist must make an inevitable compromise between missing diagnoses (False Negative, FN) and false alarms (False Positive, FP). Whenever the FP is having a greater impact, the radiologist will try to maximize the precision and whenever the FN is important, the radiologist will try to maximize the recall.

Signal theory suggests that the observer applies a gain maximization function to set the criterion according to the desired goal. Indeed, in radiology and medicine in general, unfortunately there is often overlap between positive images corresponding to the ill state and so-called negative images corresponding to the non-ill state with a normal distribution of patients. This phenomenon is expressed simply by contingency tables commonly used in medicine to evaluate diagnostic test effectiveness (**Figure 4**).

In oncology, the physician is torn between a conservative objective (i.e., avoidance of biopsies and death at the cost of missed cancers), and a non-conservative objective (i.e., not missing any cancer, even if invasive diagnostic procedures are required). The first case minimizes false positives (statistical type I error) and the second case minimizes false negatives (statistical type II error).

The decision criterion is determined to maximize the gains according to the objective that the radiologist has set. It is therefore variable according to the individual and the context.

## 4 The DAC classification of radiological errors

The Kim and Mansfield classification is the most widely accepted classification for error types in radiology (4). Based on a retrospective evaluation, this classification determined 12 error types according to their cause. We feel that this classification is imperfect because it does not fully cover the chronological nature of the causality principle.

**Figure 5:**
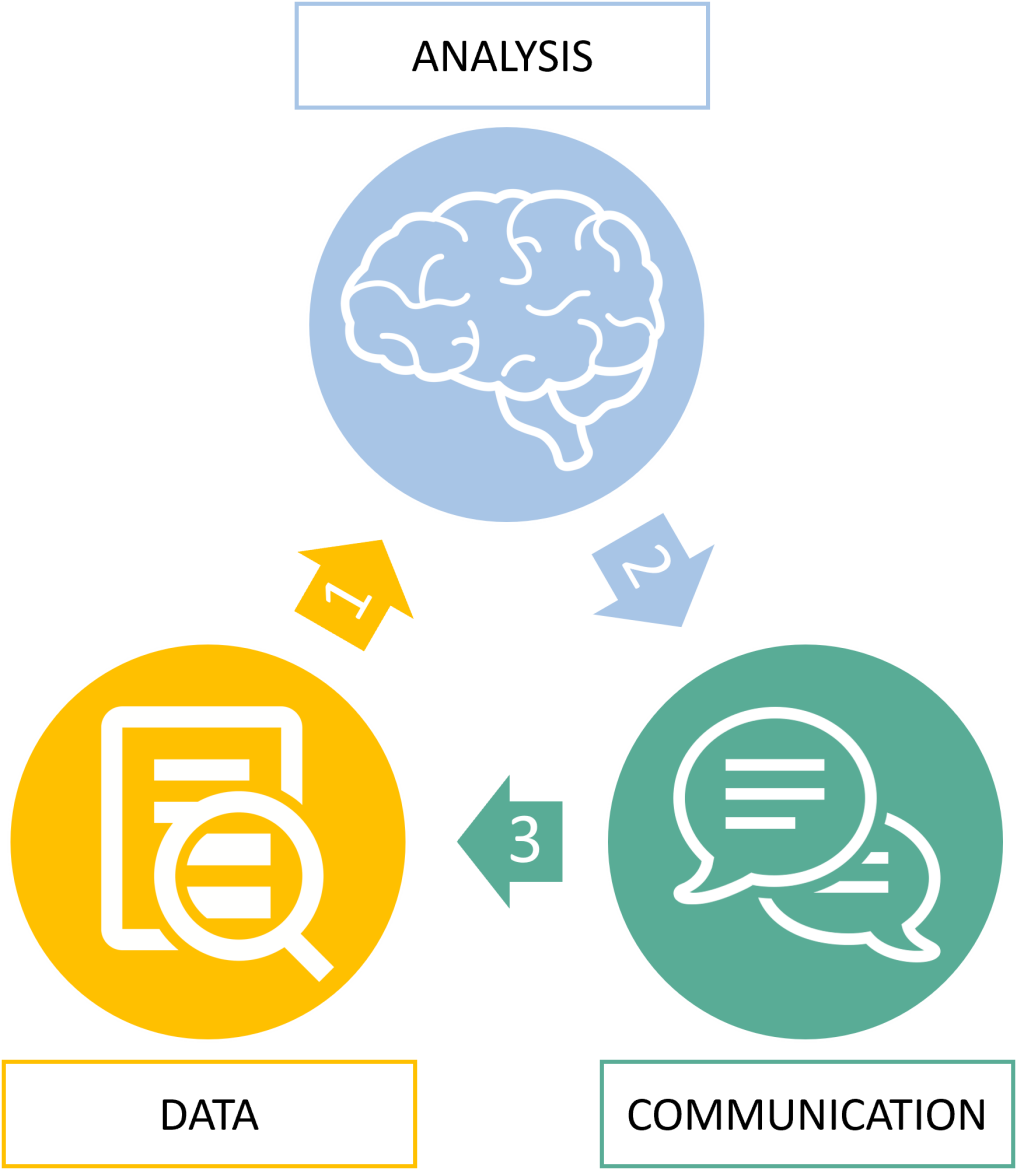
Root cause analysis of radiological errors. The virtuous circle of information during imaging diagnostic from input data (D) to data analysis (A) to communication of output data (C).

We therefore propose a simpler error classification with a simpler approach that can be illustrated with the information flow (**Figure 5** and **Table 1**).

**Table 1:** The “DAC” classification of errors in diagnostic imaging.

ERROR TYPE	INFORMATION PROCESSING	DEFINITION	KIM & MANSFIELD TYPE	%
**D**	Data Collection of and Meta-information	Failure to collect pixel and non-pixel information in respect with the good practices	Technique	2
			Prior examination	5
			History	2
			Location	7
**A**	Analysis (A1): Detection	Failure to see a retrospectively visible finding (*Under-Detection)*	Underreading	42
			Satisfaction of search	22
			Satisfaction of report	6
	Analysis (A2): Characterization	Failure to recognize the clinical significance of an identified finding *(Misclassification)*	Overreading (complacency)	1
			Faulty reasoning	9
			Lack of knowledge	3
**C**	Communication of the Analysis Result	Failure to communicate diagnostic imaging results appropriately (to the physician or the patient).	Poor communication	0

The incidence of error types is reported according to the original source of the Kim & Mansfield article (4).

This higher-level classification advantageously integrates the previously detailed processes of “information to decision” through the acquisition of information (D for data), its analysis using cognition (A for analysis) and the communication of this analysis (C for communication). We separate errors according to this DAC approach:

1. Error type D: Data or meta-data related errors
2. Error type A: Analysis through cognition related errors
3. Error type C: Communication of diagnostic results related errors

### 4.1 Error related to the data (Type D)

The first step of a radiological examination in oncology is to collect the information necessary to understand and frame the problem. It is important not to corrupt this information processing. Unfortunately, as every radiologist knows, in our practice several differential diagnoses share the same image findings (18).

Clinical information is useful to contextualize visual analysis and this meta-information improves the knowledge of what was previously named as the “prior”. This type of information is furthermore necessary as it can indirectly influence the image information when used to adapt the acquisition protocol and field of view. We also mentioned above that it influences the maximization criteria. This step of information processing is therefore critical. Unfortunately, appropriateness of imaging referral is sometimes not justified with impact on unnecessary or wrong examination (21).

**Figure 6:**
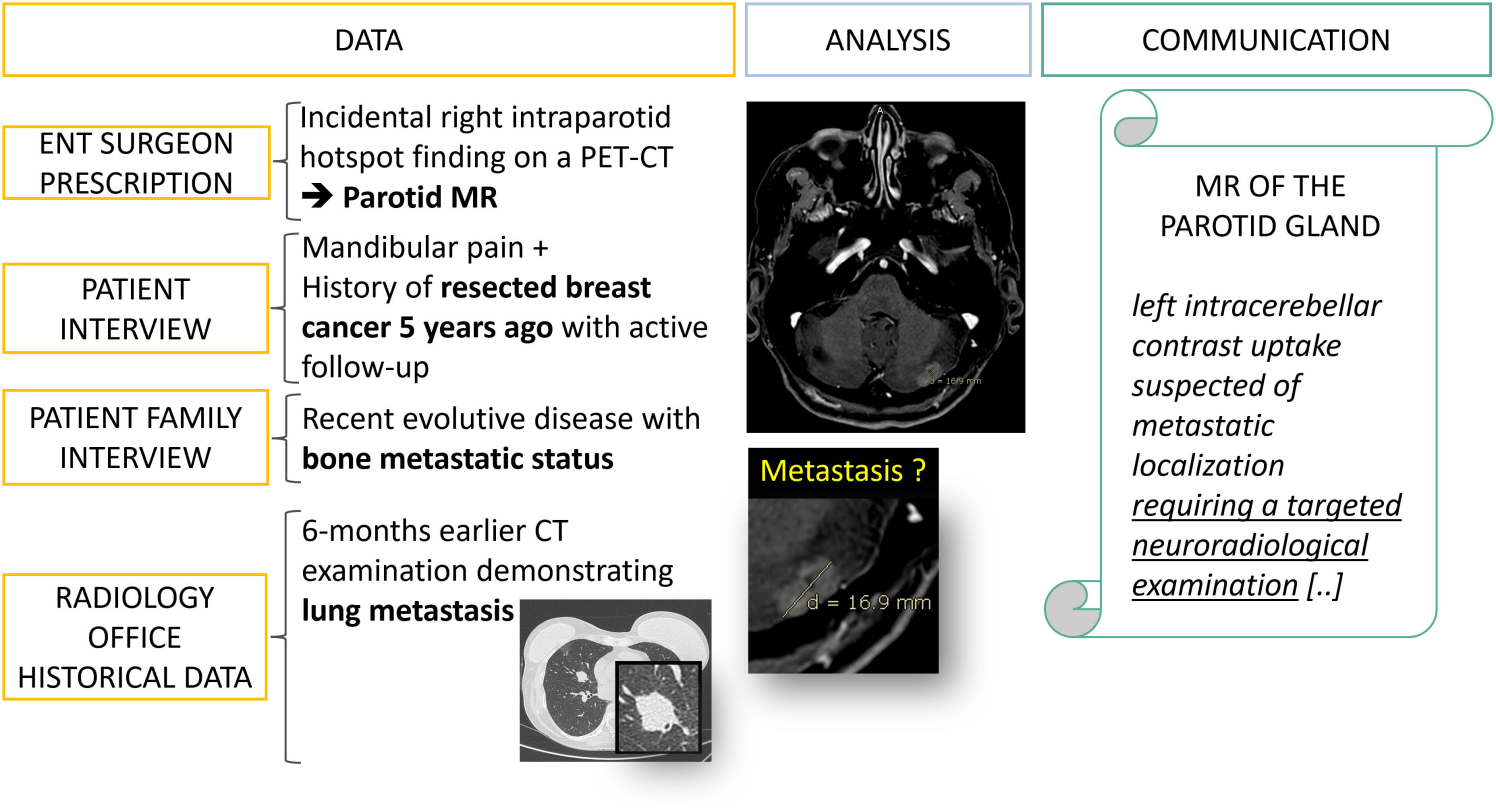
Data collection: from fragmented information to diagnostic orientation. A 50 year’s old patient is referred to a radiology office for an MRI of the parotid gland with a prescription from an ENT surgeon which mentions an incidental finding on a PET-CT. It is only after interviewing the patient, his family and querying the PACS that an evolutive metastatic cancer background status is revealed.

**Figure 7:**
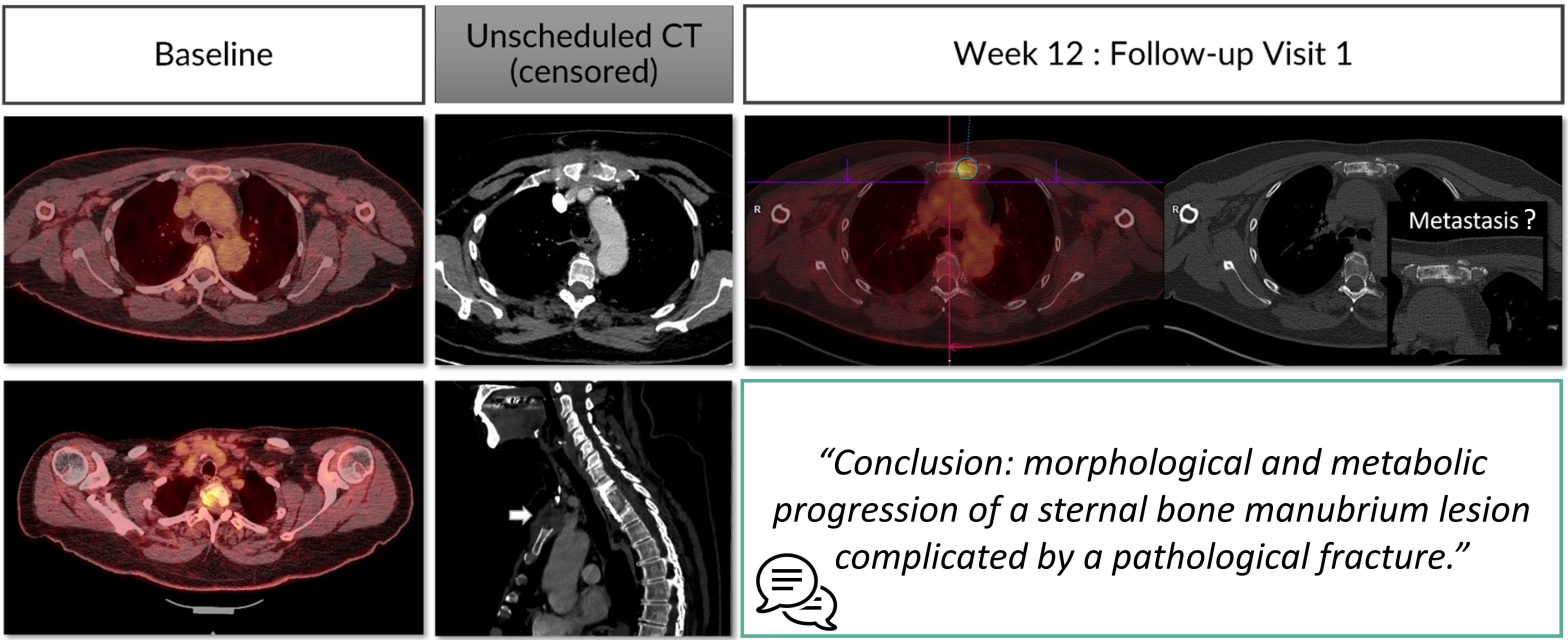
Type D error: misinterpretation linked to lacking prior information. A 53 year’s old patient is referred for a melanoma cancer follow-up with PET-CT. At the first follow-up visit, the radiologist was not aware of the metastatic vertebral stabilization with anterior sternotomy. This censored information misled the radiologist into finding a sternal lytic lesion that he thought was a metastasis.

In practice, meta-information is often available if the radiology team makes the necessary effort to retrieve it from patients, doctors, and family (**Figure 6**). Indeed, for our field of interest, it is crucial to make comparisons with historical examinations, to know the surgical interventions already performed and to question the patient if an image is confusing (**Figure 7**).

In everyday practice, it should be remembered that this type of error would be considered as a fault error since the radiologist does not have an obligation of result but rather an obligation of means. All necessary means must therefore be put in place to recover all the required information for interpretation, even if this is never easy.

The centralized clinical trial illustrates the importance of “prior” information to contextualize image analysis. Indeed, the censorship of some of the site information during blinded independent review often led to mistakes in the selection of targets for RECIST assessment with well-known benign lesion in the liver suspected to be a metastasis (22, 23).

Ultimately, the pixel-information once the examination is performed might also be corrupted if the technic of the examination was not performed correctly. It is important to provide the best technic of acquisition to avoid confusing artefacts for the analysis. If one is not satisfied with the technic of an examination, it should be reperformed with the scope to help a better-informed decision.

### 4.2 Error related to the analysis (Type A)

This type of error is also named “cognitive error” as it is linked to the cognition framework described previously (**Figures 3**, **4**).

Radiological interpretation corresponds schematically to a visual search task for significant abnormalities in one or several medical images. For didactic purposes, the task can again be broken down chronologically into two steps: detection then characterization (24).

Under-performance can either be a result of under-detection or a properly detected, but misinterpreted finding (25).

#### 4.2.1 Under-detection (Type A1)

**Figure 8:**
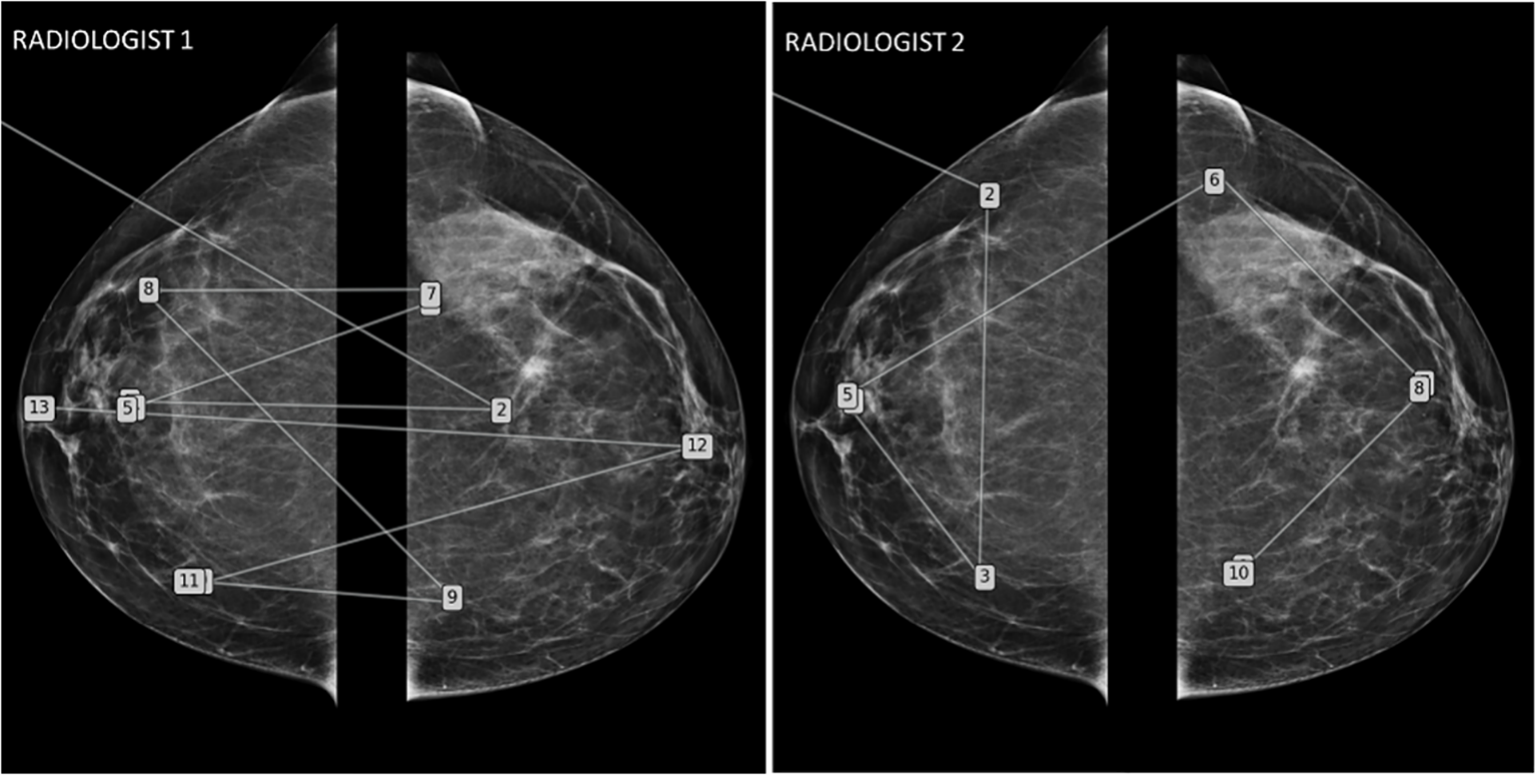
Mammography eye-gaze scan path assessments strategies. This figure illustrates two different exploration strategies driven by top-down processes learned during their training. The radiologist 1 adopted a comparative “quadrant analysis” from outer to inner quadrant to retrieve information from the comparative analysis with a “Z” shaped scan path. Radiologist 2 adopted a “side analysis” exploring first the entire right breast then the left breast.

**Figure 9:**
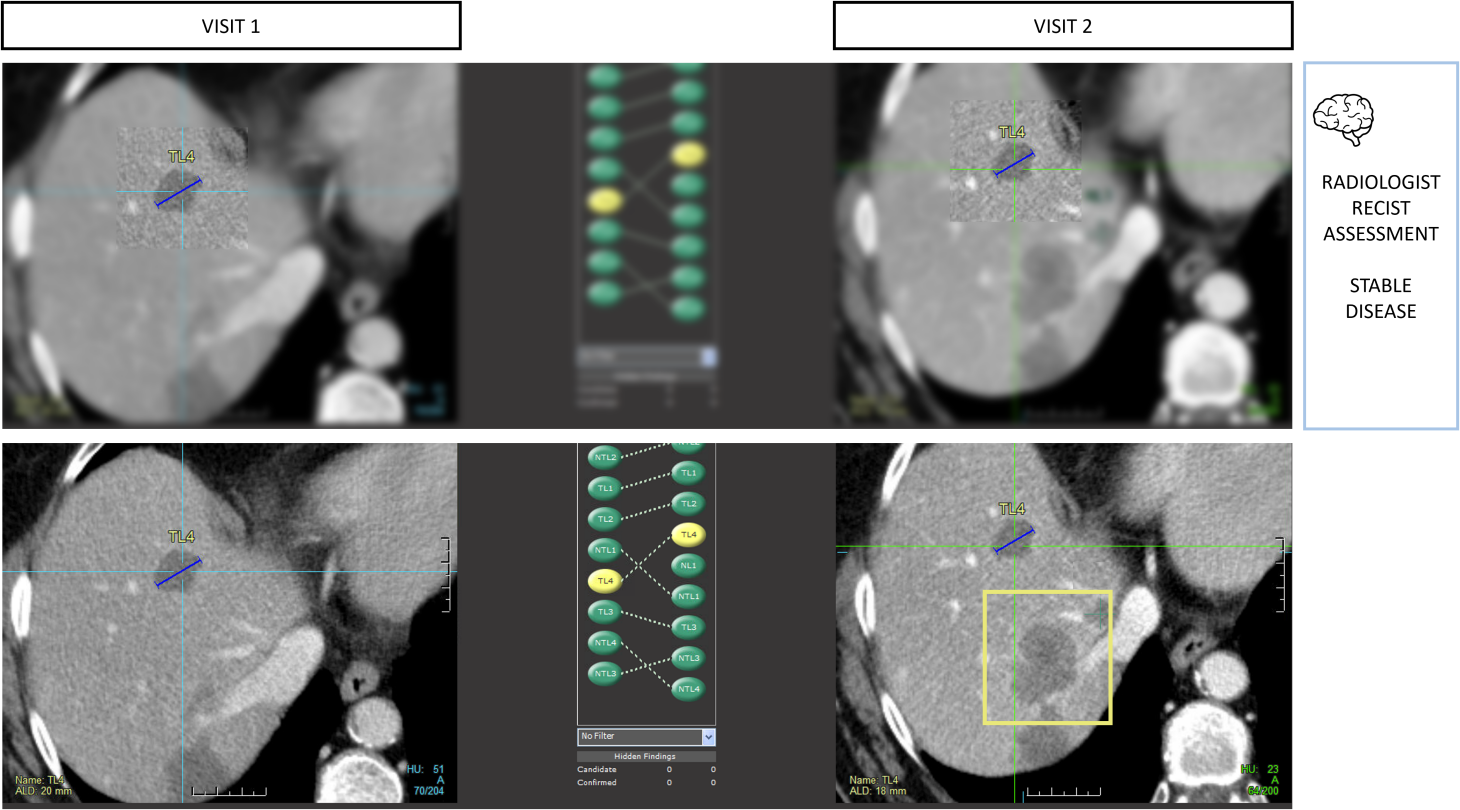
Type A1 error: inattentional blindness bias and missed lesions. During RECIST 1.1 assessments, radiologists are vulnerable to attention bias. In this example, the radiologist measured 4 targets in total including 2 in the liver using tumor tracking software. During the measurement phase, the radiologist activates his macular vision which offers the best spatial resolution in a restricted area of the image and contributes to the off-field detection error. Those targets were stable, so progression was not expected, leading the reader to miss the large new lesion (bounding box) despite it being visible on the same slice level.

During detection, the two determining factors are the visibility of the lesions and the radiologist’s detector performance:

- *Concerning the visual stimulus* associated with the lesion, its visibility can be estimated by the signal-to-noise ratio. In addition, its cognitive integration can be conducted through two processes:
  - Bottom-up processes depend almost entirely on the information perceived, and therefore little on the assumptions or expectations of the perceiving radiologist.
  - Top-down processes based on integration of the previously learned information on this perceptual information. They are high-level cognitive processes and control the sensory information from knowledge and experience.
- *Regarding the performance of the human detector*, this depends on the analysis method and the level of attention.
  - The visual analysis strategy varies between radiologists (**Figure 8**) depending on their knowledge and experience. It has been described that there are significant differences in the exploration of a CT volume or mammograms between radiologists and that these differences correlate with different detection efficiencies (26, 27).
  - Attention to the task is also a factor contributing to variability. It has been documented that radiologists may miss abnormalities that are visible retrospectively, either because of a drop in attention level, or because of a shift in attention. Cognitive biases can interfere with the radiologist’s attention and lead to non-detection errors.
    ▪ “Satisfaction bias” is well-documented in radiology (28). It refers to a drop in attention after the discovery of an abnormality. Hence, it is responsible for the non-detection of additional abnormalities. In oncology, this is exemplified by detection of one pathological finding but miss detection of multifocal lesions.
    ▪ “Inattentional blindness” bias is also documented in radiology. It refers to attention locked in a top-down process that prevents the detection of unexpected anomalies (**Figure 9**). This phenomenon is illustrated by the popular article entitled “The invisible gorilla strikes again[ … ]” and others have reproduced the phenomenon (29, 30).

#### 4.2.2 Misinterpretation (Type A2)

The characterization process involves the radiologist’s judgment. Once a finding is detected, the question of diagnosis arises. Misinterpretation is rarely due to the responsible radiologists’ lack of knowledge. In documented series, this represents around 10% of errors (4, 24, 25). Frequently, it is to do with the functioning of the brain, that uses a heuristic strategy for information processing. These heuristics reduce the brain’s workload at the cost of systematic errors. These cognitive biases are widely documented in radiology and oncology, with anchoring, confirmation and availability biases most frequently encountered (31).

**Figure 10:**
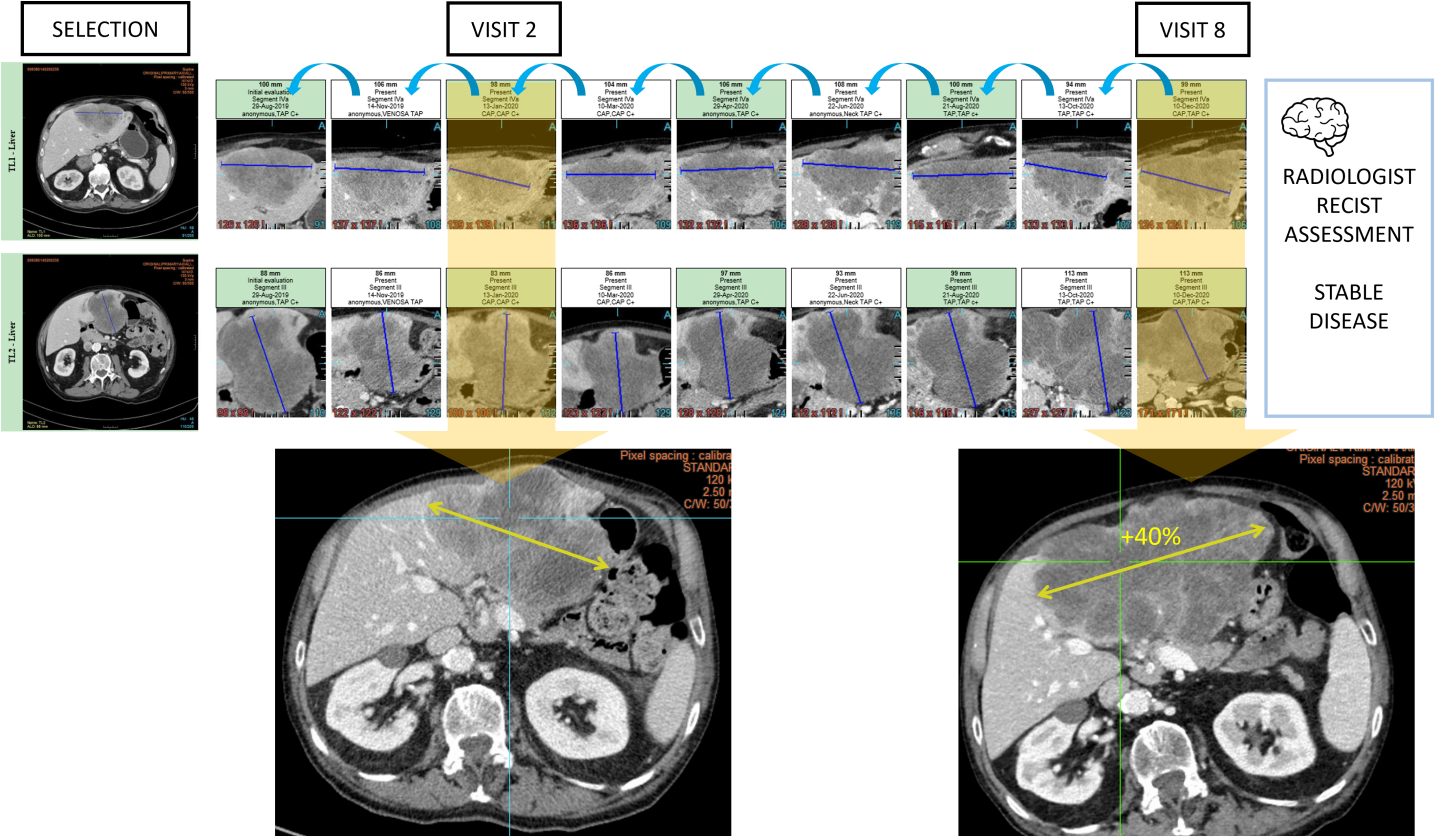
Type A2 error: anchoring bias and measurement distortion. During oncologic RECIST 1.1 assessments, radiologists are vulnerable to anchoring bias. In this example, the radiologist chronologically assessed 9 examinations and measured 2 targets in the liver to conclude stable disease. However, the same measured lesion when reviewed from visit 2 with the visit 8 clearly demonstrated a progression.

Oncological follow-up is vulnerable to anchoring bias when the analysis is conducted in a sequential manner. In such a setting, we observe that the radiologists tend to confirm the previous measurement result while this measurement operation is reputed to be non-subjective (**Figure 10**).

### 4.3 Error related to the communication of report (Type C)

In oncology, radiological evaluation is central for diagnosis and treatment decisions. Tumor follow-up criteria are now used as a decision-making tool not only for clinical trials, but also for routine use.

Communication of results is often delicate because it is aimed at both the patient and the physician, sometimes with a slightly different objective.

The communication medium is the written report. In routine, this error is difficult to trace and the Kim and Mansfield analysis probably underestimates its frequency (4). However, in clinical trials, non-conformity of reports is documented as a frequent deviation with 55% (32).

**Figure 11:**
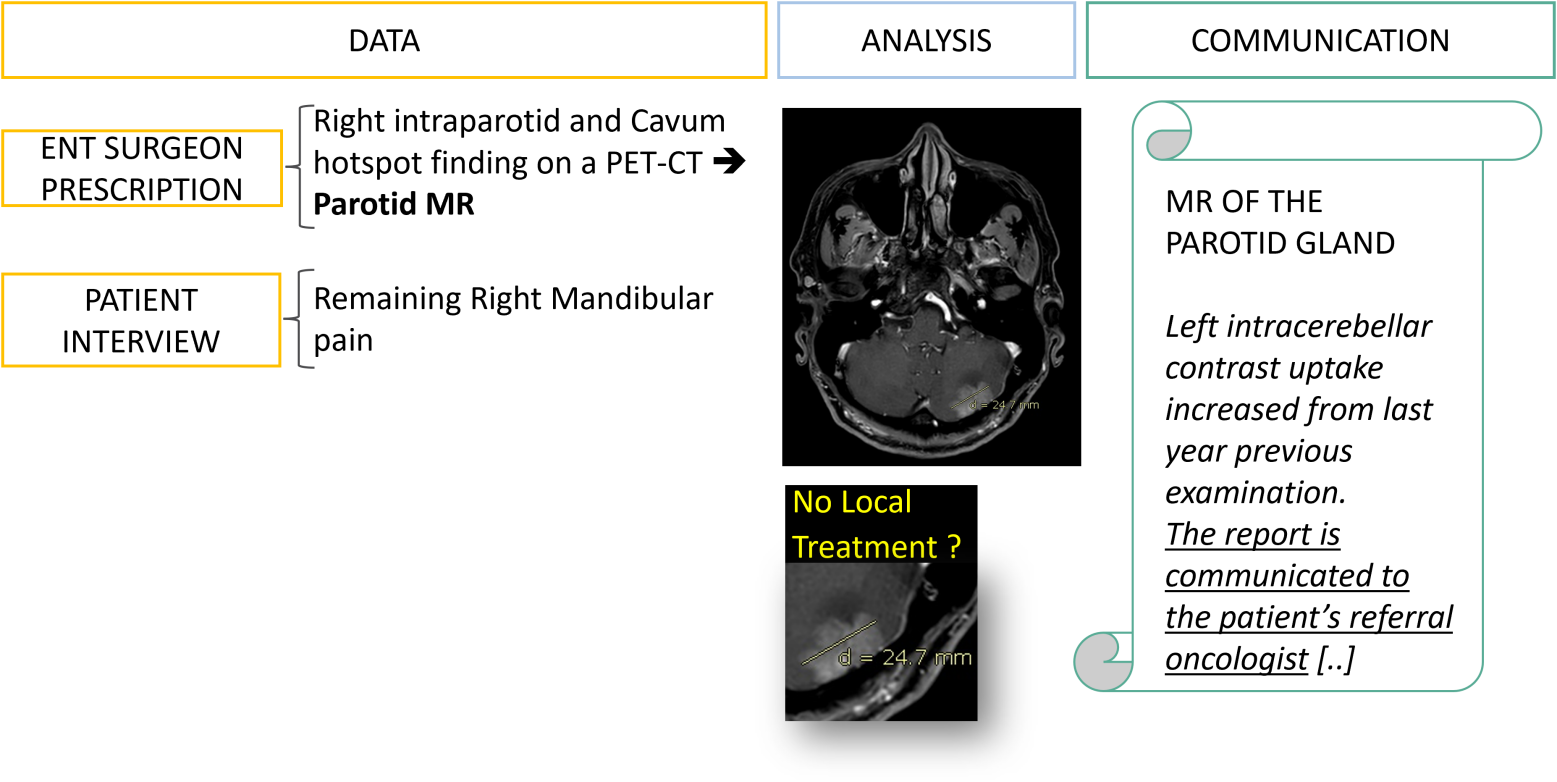
Type C error: delayed management of a brain metastasis linked to miscommunication. At the first follow-up visit the data collection allowed the office-based working radiologist to detect and suspect a brain metastasis of an evolutive breast cancer while the hospital-based ENT surgeon prescription was not mentioning any specific history of cancer (**Figure 6**). However, one year later the patient came to the same office to perform the same examination and the brain lesion had increased. After investigation, the initial report has been received by the ENT surgeon who assumed that the oncologist team was already aware of the brain metastasis. This miscommunication led to a complaint from the patient about delayed management of the brain lesion.

These type C errors are no less impactful and lead to bad decisions because of incomplete, false, or misunderstood information (**Figure 11**) (11).

## 5 Discussion

Radiology errors are addressed to prevent occurrence of adverse events. Since error is statistically embedded in the above predictive decision-making model, the objective is to minimize the individual related sources of error previously outlined.

**Figure 12:**
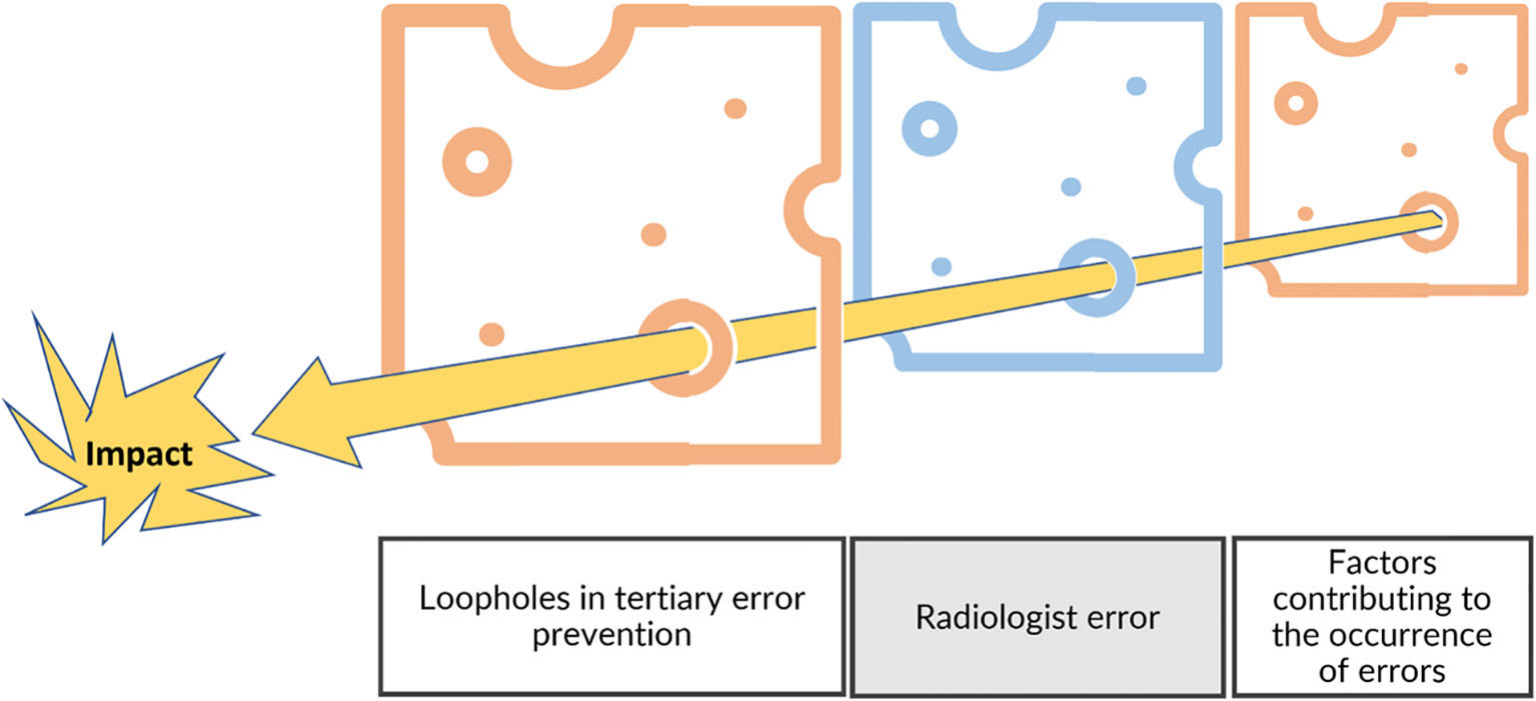
Swiss cheese model and risk management. In addition to the individual level, the risk management model needs to include the environment by considering the factors contributing to errors and the fail-safes that were not efficient (tertiary prevention).

However, it is necessary to consider radiological interpretation in its global environment to differentiate the control strategy for the individual’s error from a systemic approach focusing on conditions and factors acting on this individual (**Figure 12**) (33).

According to the pareto principle, in relation to the 3 classes of errors described above, we can propose a toolbox trying to address the most frequent contributors (**Table 2**). Cognitive type A errors dominate and represent up to 80% in reported series (4, 25).

**Table 2:** Individual risk management.

ERROR TYPE	PREVENTION
**D**	• Diversify the sources of collection of clinical information (patient, accompanying person, secretary, technician) • Adapt the protocol to the region of interest • Retrieve the imaging history • Postpone the analysis if there is lack of information • Contact the referring physician (obligation of means)
**A1**	• Systematic analysis of what is not related to the specific question • Checklist (we only find what we are looking for) • Avoid the satisfaction bias with systematic search for additional findings if an anomaly is detected
**A2**	• Keep theoretical knowledge up to date (Continuing Medical Education) • Postpone the decision with a reconsideration of the first interpretation to confirm the first judgment. • Awareness of the most common cognitive biases
**C**	• Know and apply of consensus-based criteria for oncologic follow-up • Consider structured reports and templates • Be comparative • Be able to communicate a notion of uncertainty

Corresponding radiologist’s toolbox to prevent errors type DAC.

Preventing cognitive errors typically requires the use of debiasing strategies, which aim to either prevent or correct the initial judgment, often formed through heuristics However, corrective strategies face practical limitations, as they require either revisiting and re-evaluating one’s initial decision or relying on a concurrent external opinion to provide a counterbalance. Both approaches are time-intensive and frequently infeasible in high-pressure environments like clinical practice, where time constraints may not allow for thorough reconsideration. Moreover, merely being “aware” of cognitive biases does not necessarily reduce their impact after they have already occurred. Research has shown that awareness alone often fails to mitigate cognitive errors because biases are deeply ingrained in our cognitive processes and operate subconsciously. Additionally, measuring individual susceptibility to cognitive biases is still an area of ongoing research (34, 35).

Then, even if type D errors are less frequent, the preventive action might be more effective in making all necessary efforts to collect the best clinical information and pixel information.

Also, regular training helps to reduce the type A errors linked to lack of knowledge errors and might also improve the quality of communication (6, 36). Large literature insists from several years on the necessity to improves the quality of reporting by improving communication skills of radiologists and using several tools such as structured reports and multidisciplinary standardized disease lexicon and classifications (37, 38).

Sources of error are multifactorial and dependent on the radiologist’s environment (39). We can consider primary prevention to reduce systemic risk factors and secondary or tertiary prevention for early correction of potential radiologist errors (5) (**Table 3**).

**Table 3:** Systemic risk management.

SYSTEMIC RISK FACTORS OF ERROR	PREVENTION
*Work Conditions*	• Adapt amount of work and time to deliver • Prevent multitasking and interruptions • Adopt a quality approach in medical practice (risk criticality and mapping with monitoring strategies, external audit) • Promote peer-review and follow-up of cases
*Workflow*	• Optimize dataflow with available previous examination and measures • Patient triage to prioritize cases needing more attention • Human assist or computer assist image post processing automations for repetitive tasks such as measurements
*Fail-safe*	• Tumor tracking software with criteria-read rules and compliance check • Second opinion ○ Second radiologist ○ Computer-aided diagnosis

Strategies to reduce risks factors of individual errors or to correct early detected errors.

We initially illustrated the cognitive feedback loop of the mistakes to create knowledge for future diagnostics. To make it possible, the working conditions should promote positive communication regarding errors, which are in fact an opportunity for improvement. Sharing errors during a regular staff meeting or peer review is a good way to dramatize the error in radiology (40, 41). However, these facilities are resource intensive, jeopardizing their feasibility (42).

Poor working conditions have been established as a risk factor for interpretation errors by several authors (43).

The number of images per minute read by radiologists has increased by 7 in this age of hyper-efficiency, driven by the digitalization that occurred in the 2000’s (43, 44). In the event of overwork, the risk of error for the radiologist increases. It has been estimated that a 2-fold increase in examination rate increases the risk of omission errors by 25% (45). We conducted a survey of 35 radiologists in the south of France, and found that 80% of radiologists interpreted >20 CT scans per 4-hour shift; some authors have shown that there is a significant increase in errors when performing more than 20 CAP-CT scans per day (46).

More than 2/3 of errors are caused by cognitive attention bias. It is reported that interconnectivity leads to multiple interruptions in the workflow that affect the radiologist’s attention (47). This multitasking distracts the radiologist, increasing the risk of error. Some authors have shown that radiologists can be interrupted every 4 to 12 minutes.

Workflow in oncology is essential because radiological analysis consists of comparing and measuring lesions repeatedly. Previous measurements must be easily available at the time of analysis by optimized equipment. Moreover, the measurement step is time consuming, and some authors propose a hybrid workflow after the baseline measurement to decrease the examination time for the radiologist without loss of quality (32). The same automatic computation for image post-processing analysis (mainly measurements) in any radiological field could greatly help to reduce workload therefore indirectly reduce attention bias linked to these mentally consuming tasks.

Furthermore, the negative predictive value of artificial intelligence could potentially read the content of images and propose a prioritization of patients with significant radiological abnormalities, allowing more time and attention to be spent on these at-risk patients compared to others, but this means to qualify and build trust into AI-triage systems (48, 49).

It is generally accepted that “two brains are better than one”. An important fact to keep in mind is the importance of the communication of one’s confidence level with second readers as this seems to be a determinant in the application of this adage (50). The second radiological opinion has been shown to be effective in several studies and this paradigm is used in centralized independent imaging readings for clinical trials (23, 51).

The second opinion can also come from a machine. The developments of artificial intelligence in detection and characterization should allow it to compete with a radiologist’s readings in the future.

Also, specifically in oncology, “tumor tracking” software can integrate the analysis rules for follow-up criteria and enable prevention of non-compliance errors during the analysis time i.e., number of targets, minimum size.

More generally, awareness about the risk control necessity to prevent errors is promoted through good practices of quality management. Imaging departments should start to implement quality assurance standards helping them to detect and correct the risks of errors (7).

Audit of their working condition and performance should also benefit to reduce individual errors by unmasking such correctable environmental risk factors (52).

## 6 Conclusion

The errors detected are only the tip of the iceberg as many of them will not have a significant enough impact to trigger a complaint. Oncology, which deals with a serious disease and regular examinations, is the indication that provides the best insight into the ins and outs of medical imaging errors.

For the sake of understanding, we proposed a threefold classification of mechanisms of error related to the information (D), the cognition (A), and the reporting (C).

However, it is important to understand that even if we tried to systematize it, the error in radiology partially escapes this systematization because it occurs in a complex and non-deterministic world. The DAC classification describes an over-simplified model, still it offers a practical means for risk management to identify and operate on drivers of errors.

The individual factors of errors are dominated by cognitive bias, but debiasing strategies seems more feasible through environmental drivers. The toolbox that we provide are generalist and non-exhaustive. At the individual level, raising awareness of preventable errors and adopting a non-blaming behavior will help to move towards quality driven practices in radiology with benefit from a sort of collective intelligence thanks to more sharing of errors and experiences. AI-machines are a hot topic of discussion regarding error with high promises addressing the quality of care more than the reduction of the radiologist’s workload (53). Humans will still be in the loop for a while and error management will not disappear soon.
